# Verbal Semantics Drives Early Anticipatory Eye Movements during the Comprehension of Verb-Initial Sentences

**DOI:** 10.3389/fpsyg.2016.00095

**Published:** 2016-02-09

**Authors:** Sebastian Sauppe

**Affiliations:** ^1^Language and Cognition Department, Max Planck Institute for PsycholinguisticsNijmegen, Netherlands; ^2^Department of Linguistics, Ruhr University BochumBochum, Germany; ^3^Department of Linguistics and Information Sciences, Heinrich Heine University DüsseldorfDüsseldorf, Germany

**Keywords:** sentence comprehension, anticipation, prediction, visual world eye tracking, Tagalog, verb-initial word order

## Abstract

Studies on anticipatory processes during sentence comprehension often focus on the prediction of postverbal direct objects. In subject-initial languages (the target of most studies so far), however, the position in the sentence, the syntactic function, and the semantic role of arguments are often conflated. For example, in the sentence “The frog will eat the fly” the syntactic object (“fly”) is at the same time also the last word and the patient argument of the verb. It is therefore not apparent which kind of information listeners orient to for predictive processing during sentence comprehension. A visual world eye tracking study on the verb-initial language Tagalog (Austronesian) tested what kind of information listeners use to anticipate upcoming postverbal linguistic input. The grammatical structure of Tagalog allows to test whether listeners' anticipatory gaze behavior is guided by predictions of the linear order of words, by syntactic functions (e.g., subject/object), or by semantic roles (agent/patient). Participants heard sentences of the type “Eat frog fly” or “Eat fly frog” (both meaning “The frog will eat the fly”) while looking at displays containing an agent referent (“frog”), a patient referent (“fly”) and a distractor. The verb carried morphological marking that allowed the order and syntactic function of agent and patient to be inferred. After having heard the verb, listeners fixated on the agent irrespective of its syntactic function or position in the sentence. While hearing the first-mentioned argument, listeners fixated on the corresponding referent in the display accordingly and then initiated saccades to the last-mentioned referent before it was encountered. The results indicate that listeners used verbal semantics to identify referents and their semantic roles early; information about word order or syntactic functions did not influence anticipatory gaze behavior directly after the verb was heard. In this verb-initial language, event semantics takes early precedence during the comprehension of sentences, while arguments are anticipated temporally more local to when they are encountered. The current experiment thus helps to better understand anticipation during language processing by employing linguistic structures not available in previously studied subject-initial languages.

## 1. Introduction

Anticipation, the prediction of upcoming events, is an important property of human cognition and it has been argued recently that brains are essentially “prediction machines” (Clark, 2013, cf. also Bubic et al., 2010). Predictive processes are found, for example, in interaction between individuals when people predict the outcome of actions performed by others (Sebanz and Knoblich, 2009) and even their movements (Kilner et al., 2004).

Anticipation is also involved in language processing. During the comprehension of spoken or written sentences, language users build predictions about the upcoming linguistic input. Words are, for example, read faster when they are predictable from the context as compared to unpredictable words (Ehrlich and Rayner, 1981). Language users may even predict the phonological form of an upcoming word: DeLong et al. (2005) found differential EEG responses when listeners encountered a determiner (*a/an*) that did not fit with the noun that they assumed will follow (“*The day was breezy so the boy went outside to fly… a kite* vs. *an airplane”*). Anticipatory processes are also found in conversation where listeners predict the end of their interlocutor's turn, in order to be able to take their own turn in a timely manner (Magyari and de Ruiter, 2012; Magyari et al., 2014).

The visual world paradigm has been used extensively to investigate predictive processes during language comprehension. In this experimental paradigm, participants see a display and hear an accompanying sentence while their eye movements are recorded (cf. Huettig et al., 2011a for a review). In a seminal visual world study, Altmann and Kamide (1999) showed that in English the lexical semantics of verbs is used to anticipate the syntactic object of a sentence by incrementally narrowing down the set of potential referents. Participants saw displays showing, e.g., a boy, a ball, a toy train, a toy car, and a cake, and heard sentences of the form “*The boy will move/eat the…”*. The verb of the sentence could either take any of the depicted things (*move*) or only one of them (*eat*) as its syntactic object. Listeners used the verb's selectional restrictions and fixated on the corresponding element in the display already before it was mentioned when the verb only allowed one object referent in this position (*eat* and *cake* in this case).

Further visual world studies substantiate the idea that sentence comprehension is highly predictive and that listeners use various kinds of information to form their expectations. Kamide et al. (2003b) showed that case marking information can be combined with semantic information from the verb in German to anticipate syntactic objects. Kamide et al. (2003a) showed that information from several constituents can be combined to predict upcoming elements in English ditransitive sentences and in verb-final Japanese sentences. Boland (2005) showed that arguments are more likely to be anticipated than adjuncts in English. Knoeferle et al. (2005) showed that listeners rapidly integrate visual information that is provided to them and that this information is used to anticipate object referents in German, even when the sentences accompanying a display describe unusual situations and therefore run counter to listeners' world knowledge.

All of these studies have in common that they investigated how information provided by sentential and visual context are integrated to predict elements that occur at the end of sentences. The already encountered input restricts language users' attention to the anticipation of the only remaining element of the sentence. Transitive verbs, such as *eat*, take two arguments and in languages with subject-initial word order (e.g., English and German), listeners already have heard one of the arguments when they encounter the verb—the point from which anticipatory eye movements are measured in most studies. Thus, listeners already have information about one argument, including its referential identity and its semantic role (in the case of Kamide et al., 2003a even about two arguments of ditransitives). Put differently, in previous studies on subject-initial languages the anticipation target has always been a single element at the end of a sentence, conflating syntactic function, word order, and semantic role.

There is thus still an open question regarding what kind of information listeners orient to for predictive processes during sentence comprehension. Do they try to anticipate referents based on syntactic function (e.g., direct object)? Alternatively, are their expectations based solely on what they expect to follow next? Or do listeners rather exploit semantic information to form expectations about the event and therefore anticipate referents carrying certain semantic roles (e.g., patient or goal)? Unfortunately, studies of subject-initial languages are not suited to answer these questions because the three different types of anticipation targets are conflated on the last noun phrase position that is usually employed to test prediction processes. Taking Altmann and Kamide's (1999) sentences, *cake* is the direct object, the patient and the word directly following the verb. Examining the prediction of this element cannot differentiate between these three types of information as the anticipation target.

Verb-initial languages offer a possibility to disentangle these various theoretical possibilities. In these languages, the verb is the first word of a sentence and information about the described event and selectional restrictions are provided upfront, potentially enabling listeners to identify referents and the semantic roles that they play. Importantly, the early position of the verb may enable listeners to anticipate upcoming arguments before any of them is mentioned. This means that all three anticipation target types are still available—prediction based on semantic roles, on syntactic functions, or on word order. In subject-initial languages, on the other hand, one argument is always mentioned before the verb.

In the following, a visual world eye tracking experiment on Tagalog will be reported. Tagalog is an Austronesian language primarily spoken in the Philippines. The experiment was devised to test what kind of information listeners anticipate in verb-initial languages upon having heard the verb.

### 1.1. Current experiment

**Figure 1:**
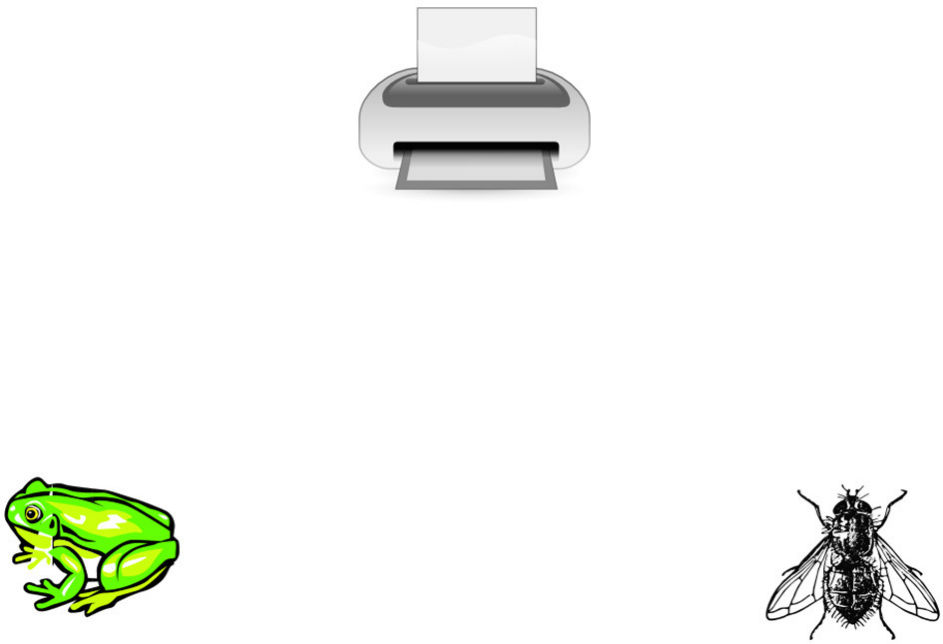
**Example stimulus**.

In the experiment described below, participants looked at visual displays depicting three potential referents (cf. Figure 1) while hearing verb-initial Tagalog sentences. Two elements in the display corresponded to the agent and to the patient of the sentences, the third element was an unrelated distractor. Participants' eye movements were recorded in order to analyze their looks to the elements as the sentences unfolded. The experiment was designed to investigate what kind of information listeners orient toward upon hearing a sentence-initial verb and what it is that they anticipate, especially when there are more possible anticipation targets than just the last word of the sentence. There are different sentence types in Tagalog that can be used to test the three possible anticipation targets; these sentence types are described in the following.

Basic word order in Tagalog is verb-initial and the verb carries voice affixes that cross-reference the semantic role of one of its arguments. This argument is marked by *ang* and will be referred to as the pivot argument. Non-pivot arguments that do not have their semantic role cross-referenced are marked by *ng*. Canonically and most frequently, the non-pivot argument immediately follows the verb and the pivot argument is realized sentence-finally (cf., e.g., Himmelmann, 2005, p. 357).

In (1a)^1^ the agent in the event (*frog*) is marked by *ang* and the verb exhibits voice morphology that signals that the semantic role of this pivot argument is agent (AV). In (1b) the patient (*fly*) is marked by *ang* and the verb signals that the pivot argument's semantic role is patient by means of different voice morphology (PV)^2^.

(1)
  a. Kakain sa umaga          ng=langaw    ang=palaka
        eat:av   in the morning npvt=fly (**P**) pvt=frog (**A**)
        “The frog will eat a fly in the morning.”
  b. Kakainin sa umaga         ng=palaka
        eat:pv      in the morning npvt=frog (**A**)
        ang=langaw
        pvt=fly (**P**)
        “A/the frog will eat the fly in the morning.”^3^

Importantly, both sentences are equally transitive. Kroeger (1993, pp. 40–48) shows with a number of syntactic tests that *ng*-marked patients in agent pivot sentences (1a) and *ng*-marked agents in patient pivot sentences (1b) are arguments of the verb. Tagalog can thus be described as exhibiting a so-called symmetrical voice system (Foley, 2008; Riesberg, 2014). This is in contrast to English where passive sentences are intransitive and the agent may only be realized as oblique.

Therefore, in sentences like (1), the initial verb provides language users with semantic information about the described event. In the context of a visual world eye tracking experiment, this might allow them to identify which referents in the visual display could sensibly be involved in the described event (e.g., a frog as the agent and a fly as the patient in sentences like in 1 or a boy and a cake as in Altmann and Kamide, 1999). Additionally, the voice marking carried by the verb provides information about the canonical order of agent and patient in the unfolding sentence. When the verb signals that the agent is the pivot (example 1a), listeners know that it will be canonically and most frequently realized sentence-finally, i.e., that the canonical order is [patient agent]. When the verb marks a patient pivot (example 1b), listeners know that the canonical order is [agent patient]. Thus, the sentence-initial verb provides listeners with information about the event from which agent and patient referents in the display can be inferred and it provides them with information about the canonical and most frequent order in which these referents will be mentioned.

Tagalog also exhibits a construction that differs from the sentences in (1) in an interesting way. Sentences in the recent perfective aspect describe events that recently happened. In these sentences the verb is not marked for voice but carries an invariant aspect marker. Thus, there is no pivot in recent perfective sentences (2) and the canonical order of arguments is [agent patient].

(2)    Kakakain pa lang ng=palaka      sa=langaw
           eat:rp      just       npvt=frog (**A**) npvt=fly (**P**)
          “A/the frog just ate the fly.”

Taken together, sentences with agent pivots, patient pivots and recent perfective sentences provide a way of investigating what kind of information language users anticipate after having heard a sentence-initial verb. The three sentence types contrast in their verb marking, i.e., whether the semantic role of a pivot argument is reflected on the verb (1) or not (2)—and if there is a pivot argument, whether it is the agent or the patient of the sentence. Additionally, the three sentence types also differ in the canonical order of the agent and patient arguments ([patient agent] for agent pivot sentences, 1a, and [agent patient] for patient pivot and recent perfective sentences, 1b and 2). Whether Tagalog listeners anticipate upcoming linguistic input on the basis of semantic or syntactic information can be investigated by comparing the comprehension of these three sentence types. It is possible to formulate differential hypotheses for each possible kind of information that may be used in anticipatory processing based on listeners' behavior during sentence comprehension. These hypotheses will be laid out in more detail in the following.

If Tagalog listeners primarily orient toward syntactic information in anticipation, they could use the semantic and morphosyntactic information provided by the verb to identify agent and patient referents and assign syntactic functions (pivot, non-pivot) to them.

A strong form of syntactically based anticipation would be the prediction of pivot arguments, i.e., that listeners anticipate the sentence-final pivot NP by already fixating on the corresponding referent in the display while or shortly after hearing the sentence-initial verb. When the verb signals that the agent is the pivot (1a), listeners should look toward the agent more after having heard the verb than when the patient is signaled to be the pivot (1b)—in which case listeners should direct their gaze toward the patient. Sauppe et al. (2013) found that in Tagalog sentence production the pivot argument plays an important role early in the planning of sentences: Tagalog speakers select a pivot at the outset of formulation in order to be able to retrieve an appropriate voice affix. If the role of the pivot argument is mirrored in anticipatory processing during sentence comprehension, fixation preferences for the agent in (1a) or the patient in (1b) are expected shortly after listeners encountered the verb.

Another syntactically based process would be the anticipation of the first-mentioned argument upon hearing the verb. Under this scenario, listeners use verbal information to identify referents and their canonical order to determine whether agent or patient will be mentioned first and will subsequently direct their gaze toward them. After having heard a verb that signals an agent pivot, listeners should direct their gaze toward the patient element in the display because the canonical word order for these sentences is [patient agent]. After having heard a verb with patient pivot or recent perfective marking, listeners should direct their gaze toward the agent referent (cf. Table 1).

**Table 1:** **Overview of sentence types; pivot arguments underlined**.

**Region**		**Verb**	**Adverb**	**NP1**	**NP2**
Agent pivot	(1a)	eat_*AV*_	in the morning	fly (**P**)	frog (**A**)
Patient pivot	(1b)	eat_*PV*_	in the morning	frog (**A**)	fly (**P**)
Recent perfective	(2)	eat_*RP*_	just	frog (**A**)	fly (**P**)

Finally, if Tagalog listeners directed their attention toward semantic roles and therefore toward the structure of the event, they should fixate on the agent in all three sentence types after having heard the verb. Agents play a prominent role in communication in general because they are initiators of events. Cohn and Paczynski (2013) propose that agents are centrally involved in building representations of events and may take early precedence during the cognition of events since they are the “heads of causal chains that affect patients” (Kemmerer, 2012). Agents are also attended to more than patients by infants (Robertson and Suci, 1980) and play a highlighted role in many grammatical hierarchies (Aissen, 1999; Lockwood and Macaulay, 2012). Given these points, it seems justified to assume that agents are the target of anticipatory processes in Tagalog if prediction was guided by semantic roles.

In the grammatical literature it has also been proposed that Tagalog exhibits a “patient primacy,” partly because sentences in which the patient is the pivot are more frequent than agent pivot sentences (cf. Latrouite, 2011 for a discussion). Theoretically, the patient could thus also be fixated preferentially after the verb was heard. However, on the hypothesis that the anticipation of semantic roles would mainly serve to construct an event representation, it seems a priori more likely that agents would be targeted for this purpose.

## 2. Experiment

### 2.1. Participants

Forty-nine students of the University of the Philippines, Diliman, participated in the experiment for payment (mean age = 18.8 years, 22 male). All of them reported being native speakers of Tagalog and speaking the language with at least one of their parents. All participants had normal or corrected-to-normal vision.

The reported experiment conforms to the American Psychological Association's ethical principle of psychologists and code of conduct (as declared by the ombudsman of the Max Planck Institute for Psycholinguistics). Written informed consent was obtained from participants at the beginning of the experiment session.

### 2.2. Materials and methods

#### 2.2.1. Materials

In the experiment, participants looked at stimulus displays while hearing pre-recorded sentences. Stimulus displays consisted of three colored line drawings that were arranged in a triangular shape (Figure 1). Line drawings either represented the agent or patient of the event described in the accompanying sentence or were distractors which were not mentioned. The position of agent, patient and distractor was counterbalanced across displays.

Displays were paired with sentences that were either intransitive or transitive. All intransitive sentences were fillers. Transitive sentences described a range of animacy scenarios in which agent and patient were humans, animals, or inanimate entities. However, scenarios in which both agents and patients were inanimate were not included. Verbs and arguments were semantically associated to varying degrees (ranging from *police car chases thief* to *owl carries bag*).

In all sentences the initial verb was followed by an adverb (*sa umaga* “in the morning,” *sa tanghali* “at noon,” or *sa hapon* “in the afternoon” for sentences as in 1 and *pa lang* “just” for recent perfective sentences as in 2). The adverb was included to increase the time between hearing the verb and the first noun phrase^4^ in order to give participants time to parse the verb and direct their gaze toward the anticipation target (cf., e.g., Kamide et al., 2003b; Mishra et al., 2012 for similar stimulus sentence structures).

Sentences were recorded by a female native speaker of Tagalog and had a neutral intonation contour so that none of the words was particularly highlighted.

Fifty-one critical displays were paired with transitive sentences which exhibited either marking of agent voice, patient voice, or recent perfective on the sentence-initial verb; agent and patient were depicted together with a distractor element semantically unrelated to the two arguments and the verb. In these displays only one element could be the agent referent and only one could be the patient referent. Seventy-nine filler displays depicted only one argument of the accompanying sentence and two distractors. The sentences were either intransitive and thus included only one argument (49 sentence-display pairs) or transitive (30 sentence-display pairs). In the latter case, one argument was mentioned but not depicted as an element in the display or two elements were possible agents or patients of the verb. Three pseudo-randomized lists were created so that each critical display occurred with one of the three sentence types in each list and at least one filler intervened between any two critical displays. For sentences describing scenarios where humans were acted on, either undergoer voice or recent perfective was used in two lists as there is a grammatical constraint against agent voice when the patient is human (Latrouite, 2011).

#### 2.2.2. Procedure

Participants were seated in front of a 17′′ laptop computer with a screen resolution of 1024 × 768 pixels. Eye movements were recorded with 120 Hz sampling rate by a SMI RED-m eye tracker attached below the computer's screen. Auditory stimuli were presented via headphones.

Trials began with the presentation of a fixation cross in the middle of the screen that triggered the presentation of the experimental display after participants looked at the cross for 700 ms. The auditory presentation of sentences started 1000 ms after the onset of the display, which stayed visible until after the end of the sentence.

After a quarter of the trials participants were asked to indicate whether all the referents mentioned in the sentence were also depicted; this was always true for the critical transitive sentences and sometimes true and sometimes false for filler sentences. Five practice trials were included at the beginning of the experiment.

The judgment task that participants had to carry out was similar to the task employed in Altmann and Kamide (1999) where participants had to indicate whether the event could apply to the picture, which was the case when all relevant referents were depicted. This kind of “look and listen” task was also employed in other visual world eye tracking studies investigating anticipatory processes (e.g., Huettig et al., 2011b). Huettig et al. (2011a, p. 154) conclude that “the listeners' eye movements during a trial of a visual world experiment reflect the direction of their visual attention, which depends both on the visual and auditory input,” i.e., listeners look at the elements in the display as they are mentioned and become relevant (Huettig et al., 2011a, p. 153). The linking hypothesis employed in the current paper is thus that listeners' gaze is a reliable reflection of their attention allocation during sentence comprehension.

Before testing, participants read instructions for the experiment in Tagalog and completed a questionnaire on their linguistic background. The whole session lasted approximately 35 min.

#### 2.2.3. Analyses

To test the hypotheses regarding possible anticipation targets outlined above, the time course of participants' fixations to agent and patient referents in experimental displays during the comprehension of the three different sentence types was analyzed.

Likelihoods of agent and patient fixations were analyzed with quasi-logistic linear mixed effects regression models (Pinheiro and Bates, 2000; Barr, 2008; Bates et al., 2015; R Core Team, 2015) in three time windows. The first time window encompassed the sentence-initial verb and the immediately following adverb (*Verb* + *Adverb* region, duration: mean = 1183 ms, *SD* = 96 ms), the second time window spanned the period during which the first argument was presented (*NP1* region, duration: mean = 703 ms, *SD* = 187 ms), finally the third time window covered the presentation of the second argument (*NP2* region, duration: mean = 815 ms, *SD* = 201 ms). To account for variations in the duration of regions across stimuli due to differing word lengths, the duration of each time window was normalized. For every stimulus, the onset of the respective region for each analysis time window corresponded to *time = 0* and the region's offset corresponded to *time = 1*. In this way, only fixations that occurred during the presentation of any given sentence region of each item were included in the corresponding analysis time windows. Fixations were aggregated into empirical logits over five consecutive bins for each analysis time window.

Time and sentence type were included as predictors in all regression models and the maximal random effects structure justified by design (that allowed the models to converge) was used (Barr, 2013; Barr et al., 2013). Significance of fixed effects was assessed using Type II Wald *F*-tests with Kenward-Roger approximation of denominator degrees of freedom (Kenward and Roger, 1997; Fox and Weisberg, 2011; Halekoh and Højsgaard, 2014). Sentence type as categorical predictor was coded with Helmert contrasts.

Trials were excluded from analyses if track-loss occurred, defined as the eye tracker having lost the participant's eyes for more than 650 ms (236 trials, 9.4%), or due to technical problems with the recording equipment (15 trials, 0.6%). Trials were also excluded if the question after a given trial was answered incorrectly; six participants that answered less than 80% of questions correctly were excluded entirely from the analyses (296 trials, 11.8%). One item was excluded from analyses because it was accidentally in the same condition in all lists. In one list, the trials from one critical display were excluded because it accidentally was presented together with a filler sentence. Three combinations of display and recent perfective sentence were discarded because they were rated as only marginally acceptable in a *post-hoc* internet-based acceptability rating study conducted with 50 Tagalog speakers from the Philippines (51 trials, 2%). Nine stimuli were excluded because the accuracy of agent recognition (given the display and the voiceless and aspect-less gerund form of the verb) was less than 10% above chance in a *post-hoc* internet-based rating study with 29 Tagalog speakers from the Philippines (322 trials, 13%). In total, 1568 trials were included in the analyses.

### 2.3. Results

**Figure 2:**
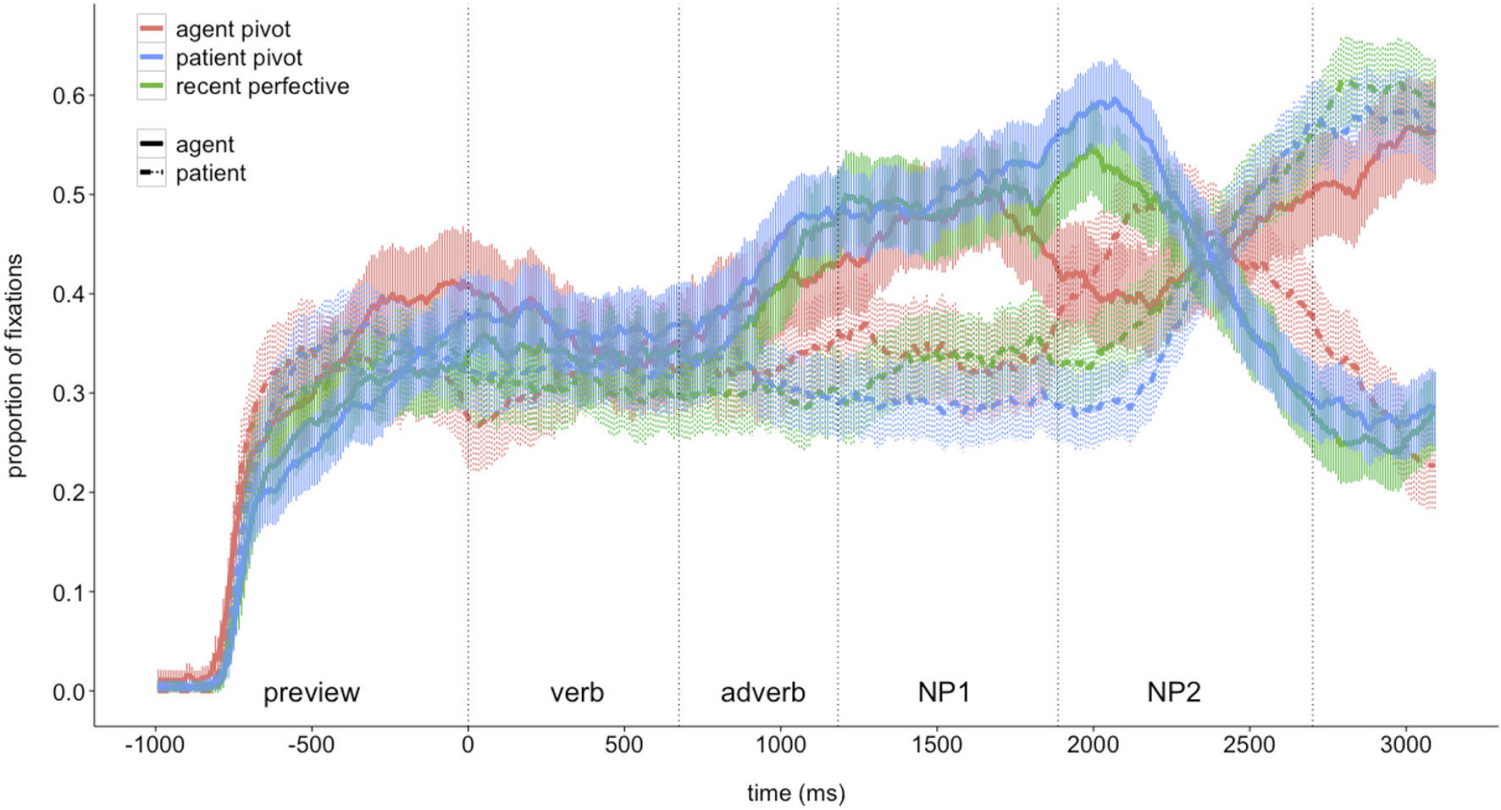
**Fixations to depictions of agents and patients during auditory presentation of three different sentence types; ribbons indicate 95% confidence intervals; vertical dotted lines indicate mean onset and offset of regions in the critical sentences (cf. Table 1)**.

The time course of listeners' fixations to agents and patients during the auditory presentation of the three different sentence types is shown in Figure 2. Visual inspection of the graph suggests that agent fixations increased during the *Verb* + *Adverb* region in all three sentence types after listeners encountered the verb. Agent fixations then continued to increase in sentences with patient voice (1b) and recent perfective marking (2) until the agent was mentioned. For sentences with agent voice marking (1a), participants' agent fixations decreased during the *NP1* region where the patient was mentioned and increased again later when the agent was mentioned during the *NP2* region. In contrast, fixations to the patient did not increase during the *Verb + Adverb* region in any of the sentence types. In sentences where the patient was encountered after the adverb (1a), participants' fixations to that referent started increasing toward the end of the *NP1* region and decreased during the *NP2* region in which the agent was mentioned. In sentences with patient pivots or recent perfective marking the patient was mentioned only sentence-finally. In these sentences, participants' fixations to the patient started to increase only toward the end of the *NP1* region and during the *NP2* region where it was mentioned.

Table 2 shows the results of the quasi-logistic linear mixed effects regression models for fixations to the agent in the three analysis time windows. During the *Verb* + *Adverb* region, only time is a significant predictor. This means that during this time window, the likelihood of agent fixations increased over time and it did so to a similar degree in all sentence types; in other words, the slope does not vary with verb marking.

**Table 2:** **Quasi-logistic linear mixed effects regression results predicting empirical logits of fixations to the agent referent in three different sentence types**.

	**By subject**				**By item**			
	**β**	***SE***	**95% Wald CI**	***F* statistic**	**β**	***SE***	**95% Wald CI**	***F* statistic**
*Verb + Adverb*								
Intercept	−0.50	0.05	−0.59, −0.40		−0.48	0.08	−0.63, −0.32	
Time	0.36	0.11	0.14, 0.57	*F*_(1, 10842)_ = 14.91^***^	0.38	0.16	0.07, 0.70	*F*_(1, 4241)_ = 6.28^*^
{PV, RP} vs. AV	<0.01	0.09	−0.17, 0.17	*F*_(2, 5754)_ = 1.17	0.03	0.07	−0.12, 0.17	*F*_(2, 18430)_ = 2.23
PV vs. RP	0.13	0.09	−0.05, 0.32		0.12	0.07	>−0.01, 0.25	
Time × {PV, RP} vs. AV	0.29	0.29	−0.28, 0.85	*F*_(2, 10546)_ = 0.67	0.17	0.18	−0.19, 0.53	*F*_(2, 43723)_ = 0.51
Time × PV vs. RP	0.15	0.19	−0.21, 0.52		0.09	0.25	−0.40, 0.57	
*NP1*								
Intercept	−0.05	0.05	−0.15, −0.05		−0.06	0.07	−0.19, 0.08	
Time	0.15	0.10	−0.04, 0.34	*F*_(1, 3879)_ = 3.41	0.23	0.09	0.04, 0.41	*F*_(1, 3471)_ = 4.86^*^
{PV, RP} vs. AV	0.20	0.10	0.01, 0.39	*F*_(2, 1257)_ = 4.72^**^	0.21	0.10	<0.01, 0.41	*F*_(2, 686)_ = 3.18^*^
PV vs. RP	0.10	0.08	−0.06, 0.26		0.11	0.12	−0.12, 0.34	
Time × {PV, RP} vs. AV	0.57	0.26	0.07, 1.08	*F*_(2, 2443)_ = 3.18^*^	0.43	0.26	−0.08, 0.93	*F*_(2, 1581)_ = 1.84
Time × PV vs. RP	0.28	0.12	−0.17, 0.74		0.26	0.27	−0.27, 0.78	
*NP2*								
Intercept	−0.22	0.05	−0.32, −0.13		−0.22	0.08	−0.39, −0.06	
Time	−0.59	0.10	−0.79, −0.39	*F*_(1, 4618)_ = 61.64^***^	−0.62	0.13	−0.87, −0.36	*F*_(1, 3317)_ = 9.91^**^
{PV, RP} vs. AV	−0.13	0.16	−0.44, 0.17	*F*_(2, 517)_ = 0.99	0.02	0.14	−0.26, 0.30	*F*_(2, 1011)_ = 8.48^***^
PV vs. RP	−0.06	0.10	−0.24, 0.13		0.01	0.14	−0.25, 0.28	
Time × {PV, RP} vs. AV	−2.22	0.22	−2.65, −1.79	*F*_(2, 4010)_ = 51.58^***^	−2.05	0.32	−2.67, −1.43	*F*_(2, 1898)_ = 23.79^***^
Time × PV vs. RP	−0.55	0.22	−0.99, −0.11		−0.40	0.28	−0.94, 0.14	

* *p < 0.05*,

** p < 0.01, and

*** *p < 0.001*.

During the *NP1* region, there was a steeper increase in agent fixations by-subjects in sentences where it was mentioned first, i.e., sentences with a sentence-final patient pivot (1b) or recent perfective marking (2). The fixation patterns associated with these two sentence types were highly similar but differed from fixation patterns observed when listeners heard sentences with an agent pivot (i.e., where the agent was heard first, 1a). This difference arose because agent fixations decreased toward the end of this time window in sentences with sentence-final agent pivots but not in the other two sentence types. By-items, the interaction of time and sentence type did not reach statistical significance. There is, however, a significant main effect of sentence type meaning that there were more fixations to the agent for sentences in which it was mentioned first, i.e., sentences with patient pivot (1b) or recent perfective marking (2), as compared to agent pivot sentences.

During the *NP2* region, agent fixations decreased in sentences with patient pivots and recent perfective marking, in which the patient was mentioned in sentence-final position, as compared to agent pivot sentences with the agent in final position. In fact, fixations to the agent in the latter sentence type increased during this time window. Additionally, there was a steeper decrease in agent fixations for sentences where the patient was the pivot argument (1b) as compared to pivot-less recent perfective sentences in the by-subjects regression model. However, this effect was not detectable in the by-items model.

Table 3 shows the results of the quasi-logistic linear mixed effects regression models for fixations to the patient in the three analysis time windows. During the *Verb* + *Adverb* region, none of the predictors reaches statistical significance, indicating that listeners' fixations to the patient did not differ between sentence types and did not change while hearing the verb and the adverb.

**Table 3:** **Quasi-logistic linear mixed effects regression results predicting empirical logits of fixations to the patient referent in three different sentence types**.

	**By subject**				**By item**			
	**$\hat{\beta }$**	***SE***	**95% Wald CI**	***F* statistic**	**$\hat{\beta }$**	***SE***	**95% Wald CI**	***F* statistic**
*Verb + Adverb*								
Intercept	−0.75	0.04	−0.84, −0.66		−0.81	0.09	−0.98, −0.64	
Time	−0.02	0.11	−0.24, 0.20	*F*_(1, 10192)_ = 0.07	0.01	0.17	−0.32, 0.34	*F*_(1, 3736)_ = 0.01
{PV, RP} vs. AV	−0.07	0.07	−0.20, 0.07	*F*_(2, 14425)_ = 0.65	−0.01	0.09	−0.19, 0.17	*F*_(2, 14124)_ = 0.15
PV vs. RP	0.04	0.08	−0.11, 0.19		0.03	0.06	−0.09, 0.16	
Time × {PV, RP} vs. AV	−0.23	0.23	−0.68, 0.22	*F*_(2, 16429)_ = 0.59	<0.01	0.20	−0.40, 0.40	*F*_(2, 27222)_ = 0.02
Time × PV vs. RP	−0.13	0.24	−0.59, 0.34		−0.05	0.24	−0.53, 0.42	
*NP1*								
Intercept	−0.70	0.04	−0.79, −0.62		−0.78	0.07	−0.92, −0.64	
Time	0.10	0.10	−0.11, 0.31	*F*_(1, 5489)_ = 0.31	0.04	0.10	−0.16, 0.24	*F*_(1, 3690)_ = 0.10
{PV, RP} vs. AV	−0.34	0.08	−0.49, −0.19	*F*_(2, 2321)_ = 10.66^***^	−0.23	0.10	−0.43, −0.02	*F*_(2, 750)_ = 3.89^*^
PV vs. RP	−0.15	0.09	−0.33, 0.02		−0.12	0.13	−0.37, 0.13	
Time × {PV, RP} vs. AV	−0.32	0.28	−0.88, 0.23	*F*_(2, 2395)_ = 0.83	−0.17	0.29	−0.74, 0.40	*F*_(2, 2015)_ = 0.20
Time × PV vs. RP	−0.12	0.19	−0.50, 0.26		−0.09	0.26	−0.60, 0.43	
*NP2*								
Intercept	−0.35	0.04	−0.44, −0.26		−0.39	0.08	−0.56, −0.23	
Time	0.56	0.12	0.33, 0.79	*F*_(1, 2228)_ = 43.27^***^	0.55	0.14	0.27, 0.83	*F*_(1, 2624)_ = 31.00^***^
{PV, RP} vs. AV	0.04	0.16	−0.27, 0.35	*F*_(2, 619)_ = 0.28	−0.02	0.14	−0.30, 0.27	*F*_(2, 849)_ = 2.97
PV vs. RP	−0.02	0.10	−0.22, 0.19		−0.04	0.11	−0.26, 0.17	
Time × {PV, RP} vs. AV	2.10	0.21	1.70, 2.50	*F*_(2, 10989)_ = 53.99^***^	1.97	0.40	1.18, 2.76	*F*_(2, 1458)_ = 18.13^***^
Time × PV vs. RP	0.47	0.18	0.11, 0.83		0.51	0.26	> −0.01, 1.03	

* p < 0.05 and

*** *p < 0.001*.

During the *NP1* region, there were more patient fixations in sentences with final agent pivots (1a) in which the patient was mentioned during that region. Listeners started to direct their gaze to the patient in this sentence type only toward the end of the time window which might explain that a main effect of sentence type but no interaction with time was found. There were no differences in patient fixations between sentences with sentence-final patient pivots (1b) and recent perfective marking (2) for which the agent was mentioned during this time window.

Finally, during the *NP2* region, there was a steep increase of patient fixations in sentences in which it was mentioned during this time window, i.e., patient voice and recent perfective sentences. Patient fixations decreased in sentences with agent pivots as they were mentioned sentence-finally. Additionally, in the by-subjects analysis, there was a steeper increase of patient fixations in sentences where it was the pivot (1b). This effect is, however, barely detectable in the by-items analysis.

To test when listeners began to direct their gaze from the referent of *NP1* to the referent of *NP2*, breakpoint analyses were performed over the corresponding analysis time windows. These analyses test for discontinuities in the linear relations (Baayen, 2008), i.e., changes of direction of the regression lines for agent and patient fixations. Participants' agent fixations began to change before the beginning of *NP2* in all three sentence types (agent pivot sentences: before the first bin of *NP2* by-subjects and by-items; patient pivot sentences: before the last time bin of *NP1* by-subjects and before the first time bin of *NP2* by-items; recent perfective sentences: before the first time bin of *NP2* by-subjects and before the last time bin of *NP1* by-items). Participants' patient fixations began to change with very similar timing (agent pivot sentences: before the first bin of *NP2* by-subjects and by-items; patient pivot sentences: before the first bin of *NP2* by-subjects and by-items; recent perfective sentences: before the last time bin of *NP1* by-subjects and by-items).

In other words, before the onset of the second argument, listeners' fixations to the agent increased in agent voice-marked sentences where it was in sentence-final position and decreased in patient voice and recent perfective-marked sentences where the patient was in sentence-final position. Similarly, before the onset of *NP2*, patient fixations began to increase in the latter sentence types and began to decrease in sentences with agent pivots.

When controlling for agent or patient animacy (humans and animals vs. inanimates) or position within the experiment (first vs. second half), or when only items that occured in all three conditions are included (i.e., excluding scenarios with human patients as sentences with agent pivots are prohibited in these configurations), a similar pattern of results emerges for all three analysis time windows. However, the different slopes for sentences with patient pivots and recent perfective sentences during the *NP2* region that were found in the by-subjects analyses for agent and patient fixations are not consistently found when these control variables were included.

Especially the similar pattern of results that was found when the position of trials in the experiment was controlled (first vs. second half) suggests that participants' behavior was not influenced by an expectation to encounter pronominalized or zero anaphora arguments (cf. Kroeger, 1993; Himmelmann, 1999). Participants seemed to be primed to encounter sentences with two full NP arguments by the practice trials at the beginning of the experiment; otherwise, some habituation over the course of the experiment modulating the effects of interest would have been expected.

Anticipatory baseline effects (Barr et al., 2011) influencing the interaction of time and sentence type are also not detectable when comparing the likelihood of agent or patient fixations during the preview and during the *Verb* + *Adverb* region (−400–200 ms relative to verb onset vs. 200 ms—*NP1* onset).

### 2.4. Discussion

The results of the current visual world experiment on Tagalog suggest that listeners used the lexical semantics of the verb to determine agent and patient referents. They directed their gaze toward the agent after they heard and recognized the verb. Interestingly, listeners focused on the agent in all three sentence types, irrespective of whether it was the pivot or not and therefore also irrespective of whether it could be expected to immediately follow the adverb or not. In contrast, while hearing the verb and the adverb, listeners did not direct their attention toward the patient.

Listeners did not seem to use information provided by the verbal morphology from which the syntactic function and the canonical position of arguments could be inferred for anticipation upon having heard the verb. If there were anticipation processes during the *Verb* + *Adverb* region based on syntactic information, i.e., if listeners either anticipated the final pivot argument or the linearly first NP, differences between sentences with agent pivots and sentences with patient pivots or recent perfective marking should have been found. Specifically, an increase in patient fixations would have been expected in sentences with agent pivots if anticipation was based on the linear order of NPs because in these sentences the patient canonically precedes the agent. Conversely, if anticipation was based on pivot status, an increase in patient fixation for sentences with patient pivots would have been expected. Yet, only fixations to the agent increased after listeners encountered the initial verb in all three sentence types.

Only after the adverb—during the *NP1* and *NP2* regions—did listeners gaze at agent and patient referents in their linear order. At least for the second argument (*NP2*), listeners seemed to anticipate the respective referent by directing their gaze toward the corresponding element before it was mentioned. Information provided by the verb and the first NP were integrated to predict the referent of the final argument. This interpretation is based on the consideration that programming a saccade typically takes approximately 200 ms (Duchowski, 2007) and there is also a lag between eye movements and the linguistic input of about the same time (Allopenna et al., 1998). Given that the slope of agent and patient fixations changed direction before the onset of the *NP2* region in most cases, it may be assumed that listeners programmed their eye movements toward the agent (1a) or the patient referent (1b and 2) already well before having heard and parsed the corresponding noun in the linguistic input.

The results of the current experiment thus indicate that early anticipation of arguments in Tagalog is based on semantic roles and that the agent of the event in particular attracted listeners' attention once enough information about the event had accumulated to allow the identification of agent and patient referents. In Tagalog, the possibilities for prediction upon encountering the verb are not already narrowed down by previous linguistic input, unlike in subject-initial languages where one of the verb's arguments, often the agent, has already been mentioned. Thus, in this verb-initial language, it appears that what is targeted by anticipatory processes is primarily the semantics of the event.

Altmann and Kamide (2007) argue for a linking hypothesis between language processing and eye movements that allows verbs to drive anticipatory eye movements based on the affordances of the linguistic input and the visual display (cf. also Tanenhaus et al., 2000). These affordances are the “properties of the possible interactions [… the depicted referents] could […] engage in” (Altmann and Kamide, 2007, p. 513). Accordingly, the presence of a frog and a fly together with the auditory presentation of “*eat”* conspire to create a representation of the event that makes the frog a potential agent and the fly a potential patient. It is this episodic fit between the semantics of the described event and the depicted referents that drives listeners' eye movements toward the agent upon having heard the verb and before the agent NP was encountered.

## 3. Conclusions

A visual world experiment on a verb-initial language was presented that was set out to test what kind of information listeners are sensitive to during anticipatory processing in language comprehension. It was found that in Tagalog, listeners focus on the agent of the event upon having heard the sentence-initial verb. The lexical semantics of the verb together with the visual display allowed them to rapidly identify agent and patient referents. It seems that listeners did not use information provided by voice marking to specifically predict the syntactic functions or the linear order of arguments right after having heard the verb.

However, later in the sentence, specifically before the second noun was encountered, listeners did integrate all available information to anticipate the corresponding referent in the sentence-final position. This finding is similar to what has been found in English (Altmann and Kamide, 1999), German (Knoeferle et al., 2005) and Japanese (Kamide et al., 2003a). Thus, users of verb-initial languages also exhibit anticipatory gazes based on the linear order of arguments. Prediction of the final NP operates on a temporally more local level and occurs right before it is encountered whereas agent anticipation after the verb is independent of its position in the sentence.

It may be concluded that there are two kinds of anticipatory processes in Tagalog: one is oriented toward the sentence-level which uses verbal semantics to identify and focus on the agent of the event, the other one operates on a local scale and integrates information from the verb and the first argument to anticipate the sentence-final argument. Anticipation of the syntactic object in subject-initial languages could then possibly be seen as an instance of the latter, temporally more local, type.

Altmann and Kamide (2007) argue that anticipatory eye movements in sentence comprehension are driven by overlapping activations between representations of the visually presented objects and conceptual representations induced by the linguistic input. The results from the current experiment suggest that verbs especially facilitate anticipation based on semantic roles. Verbs provide event semantics to which potential referents in the visual display can be associated based on their affordances. Anticipatory eye movements might reflect listeners' knowledge about the dynamics of events in the world and are therefore not only reflecting “unfolding language [… but] an unfolding (mental) world” (Altmann and Kamide, 2007, p. 515).

One possible interpretation of the findings from Tagalog is thus that language users may engage in simulation-based anticipation when processing verb-initial sentences. Huettig (2016) suggests that there are several anticipatory mechanisms in language comprehension. One of these mechanisms engages perceptual simulation of events in order to predict their outcome and the linguistic structure with which they will be represented. Moulton and Kosslyn (2009) argue that simulation and mental imagery play a vital role for the prediction of future states of the world. Cohn and Paczynski (2013) propose an agent saliency principle that renders agents more prominent than patients in the processing of events in general (cf. also Kemmerer, 2012; Bornkessel-Schlesewsky and Schlesewsky, 2013b). Upon having heard the sentence-initial verb, Tagalog listeners identified the agent referent and might have focused on it because it was the initiator of the described event and was therefore necessary to build an event structural representation and to form expectations about the remainder of the sentence. The results of the current experiment are consistent with the idea that Tagalog listeners mentally simulated the event described by the verb after having encountered it (Pulvermüller, 2005). Agents might attract the most attention during the mental simulation of events because they function as cognitive attractors as they are the instigators of these events (Bornkessel-Schlesewsky and Schlesewsky, 2013a) and because the representation of agents and their actions is probably evolutionary ancient as it is already present in infants (Spelke and Kinzler, 2007).

The current findings are also in accord with approaches to sentence comprehension that assume agent identification to be an early processing step. Bornkessel and Schlesewsky (2006) posit that listeners try to identify the agent as quickly as possible. Many studies also show that sentences in which the agent precedes the patient are easier to process (Schriefers et al., 1995; Traxler et al., 2002; Ferreira, 2003; Wang et al., 2009, inter alia).

Interestingly, the prominence of the agent role in comprehension processes in Tagalog has its reflexes in grammar, too. Schachter (1995) shows that both pivots and agents are privileged in different syntactic constructions (cf. also Schachter, 1976; Foley and Van Valin, 1984). Riesberg and Primus (2015) argue that even in Tagalog's symmetrical voice system, where verbs are morphologically marked for agent as well as patient pivots, agents have a special grammatical status. For example, agents are always binders of reflexives, independently of their syntactic status (Schachter, 1977). Thus, although there is no grammatical preference for agents as pivots—and patient pivots are in fact more frequent in Tagalog texts—, agents seem to take a prominent role in both processing and grammar. This is surely to be attributed to their centrality for event cognition.

Focusing on a different kind of simulation than the mental simulation of events described above, Pickering and Garrod (2013) proposed that anticipation in language comprehension emerges through prediction by (linguistic) simulation of production processes (cf. also Pickering and Garrod, 2007; Dell and Chang, 2014). Under this view, listeners use the linguistic input that they have encountered at any given point in time to build an impoverished forward production model of what they would say if they were the speaker, just as people construct forward models of motor commands (Wolpert et al., 2003). The output of this forward production process is then matched against what was actually heard. Thus, the production system would be routinely employed during comprehension by covertly imitating the speaker's behavior in order to build expectations about the following linguistic material before it is encountered.

Based on eye tracking evidence from sentence production in Tagalog, it seems that the current experiment does not directly support this view. Sauppe et al. (2013) show that in early stages of Tagalog sentence production the pivot argument plays a prominent role—irrespective of its semantic role. In a picture description experiment, speakers preferentially fixated the character that was to become the pivot argument before uttering the sentence-initial verb in order to aid encoding the morphological marking. By contrast, the current experiment found that during sentence comprehension in the presence of visual stimuli, listeners directed their attention toward the agent irrespective of which argument was the pivot of the sentence. Taken together, these results suggest Tagalog speakers and listeners prioritize the processing of distinct kinds of information during the early stages of sentence encoding and decoding.

In other words, during early phases of sentence production Tagalog speakers focus their attention on pivot arguments. During comprehension, on the other hand, Tagalog listeners focus on the agent of the event early after having heard the sentence-initial verb. This suggests that different processes may be at play and that listeners did not immediately build a forward production model of the unfolding sentence to predict upcoming words. If this would have been the case, agent and patient fixations in sentences with agent pivots and patient pivots should have differed based on the differential semantic roles of the pivot arguments. When producing a sentence, Tagalog speakers need to choose a pivot argument and encode the relevant information in form of voice affixes on the verb and case markings on the arguments. When comprehending a sentence, language users do not have to engage in choosing a pivot argument themselves. They can thus rather concentrate on verbal semantics in order quickly build a representation of the described event.

Nevertheless, effects of agent prominence can also be detected in production processes in Tagalog as the planning of sentences with agent pivots exhibits lower cognitive load requirements than the production of sentences with patient pivots (Sauppe, submitted).

It may be noted that it can not be excluded that local thematic priming between verb and arguments had an influence on listeners' gaze behavior. Kukona et al. (2012) found that anticipatory fixations in a visual world sentence comprehension experiment on English were influenced by semantic priming from verbs when there were strong associations between the verb and its arguments (e.g., *arrest* together with *policeman* and *crook*). Most notably, upon having heard the verb, listeners looked at potential agent referents even if they were not mentioned. It is to be determined in future studies whether these results can also be explained by the relative saliency of agents in the build-up of event structural representations and in how far priming effects influence early agent fixations in Tagalog.

In general it can be concluded that the structure of the input guides the uptake and integration of visual and linguistic information. The current study shows that in addition to selectional restrictions and other structural information (Altmann and Kamide, 1999; Kamide et al., 2003a; Boland, 2005), the semantic roles of event participants might also be targeted by anticipation processes. Verb-initial languages might even favor the anticipation of semantic roles because information about the event is presented at the very beginning of an unfolding sentence and neither agent nor patient role are already (lexically) filled upon encountering the verb.

Altmann and Kamide (1999) propose that any information available to the listener is used to anticipate upcoming elements of an unfolding sentence. The results of the current experiment on Tagalog comprehension support this view. As soon as relevant information was available, listeners used selectional restrictions to identify the verb's arguments. Later on, accrued information about the event and the already encountered words was used to anticipate the final noun phrase of a sentence. Interestingly, upon having heard the verb, language users first directed their attention toward the agent, the instigator of the described event, independently of its syntactic status and its position in the sentence.

Going beyond the findings of previous visual world studies on subject-initial languages, the current experiment employed constructions in which the influence of event semantic information and syntactic information could be dissociated. It was shown that it was semantic information that was targeted early by predictive processes although syntactic information was also prominent and became relevant later. During the comprehension of languages with subject-initial word order, predictive processes on the basis of semantic roles might also operate. As mentioned in the introduction, when anticipating sentence-final syntactic objects, listeners could specifically predict the patient referent based on its role in the event described by the verb (cf. Kukona et al., 2012). This, however can not be observed as directly as in verb-initial languages because for the anticipation of sentence-final objects, semantic and syntactic information cannot be disentangled.

Tagalog has a relatively simple verbal morphology in the sense that only the semantic role of one of the arguments is cross-referenced. Future research should address whether the richness of verbal morphology has an influence on anticipatory processes. It could be possible that, e.g., person or number marking of pivot and non-pivot arguments (or subject and object for this purpose) on an initial verb triggers different anticipatory processes because more grammatical information about arguments is provided early.

To date, there are only few studies on online language processing in verb-initial languages (most notably Sauppe et al., 2013; Norcliffe et al., 2015b; Wagers et al., 2015). These languages provide valuable means to put to test processing theories and hypotheses that were developed based on the small set of languages that is usually used in psycholinguistics (such as English, German, Dutch or Japanese; cf. Jaeger and Norcliffe, 2009 on the most studied languages in sentence production research). Making use of the grammatical diversity of the world's languages will help to refine psycholinguistic theories and to uncover processes that cannot be observed by experimentation on the “usual suspect” languages (Levinson, 2012; Norcliffe et al., 2015a).

## Author contributions

The author confirms being the sole contributor of this work and approved it for publication.

### Conflict of interest statement

The author declare that the research was conducted in the absence of any commercial or financial relationships that could be construed as a potential conflict of interest.

## Footnotes

^1^The following abbreviations are used in the current paper: A, agent; av, agent voice; npvt, non-pivot argument; P, patient; pv, patient voice; pvt, pivot argument; rp, recent perfective aspect.

^2^Tagalog also exhibits a variety of other voice forms where, e.g., the instrument, the beneficent or the location of an event is the pivot and has its semantic role cross-referenced at the verb (e.g., Schachter and Otanes, 1972; Himmelmann, 2005).

^3^Differences in the definiteness of agent and patient in the translations arise due to constraints on interpreting the *ang*-marked argument as specific (Adams and Manaster-Ramer, 1988, cf. also Latrouite, 2015).

^4^Strictly speaking, the arguments are expressed by determiner phrases headed by the markers *ng, ang* and *sa*, which define the referential meaning of the phrases. Content words are not sub-classified for syntactic categories in Tagalog and therefore there are no noun and verb classes (Himmelmann, 2008). For the sake of simplicity, however, the term *NP* will be used in this paper, following Himmelmann (2005).
